# Height, social comparison, and paranoia: An immersive virtual reality experimental study

**DOI:** 10.1016/j.psychres.2013.12.014

**Published:** 2014-08-30

**Authors:** Daniel Freeman, Nicole Evans, Rachel Lister, Angus Antley, Graham Dunn, Mel Slater

**Affiliations:** a Department of Psychiatry, University of Oxford, UK; b Oxford Health NHS Foundation Trust, UK; c Centre for Biostatistics, Institute of Population Health, University of Manchester, UK; d Department of Computer Science, University College London, UK; e Institució Catalana de Recerca i Estudis Avançats (ICREA), University of Barcelona, Spain

**Keywords:** Paranoia, Height, Social rank, Virtual reality

## Abstract

Mistrust of others may build upon perceptions of the self as vulnerable, consistent with an association of paranoia with perceived lower social rank. Height is a marker of social status and authority. Therefore we tested the effect of manipulating height, as a proxy for social rank, on paranoia. Height was manipulated within an immersive virtual reality simulation. Sixty females who reported paranoia experienced a virtual reality train ride twice: at their normal and reduced height. Paranoia and social comparison were assessed. Reducing a person's height resulted in more negative views of the self in comparison with other people and increased levels of paranoia. The increase in paranoia was fully mediated by changes in social comparison. The study provides the first demonstration that reducing height in a social situation increases the occurrence of paranoia. The findings indicate that negative social comparison is a cause of mistrust.

## 1 Introduction

**Fig. 1:**
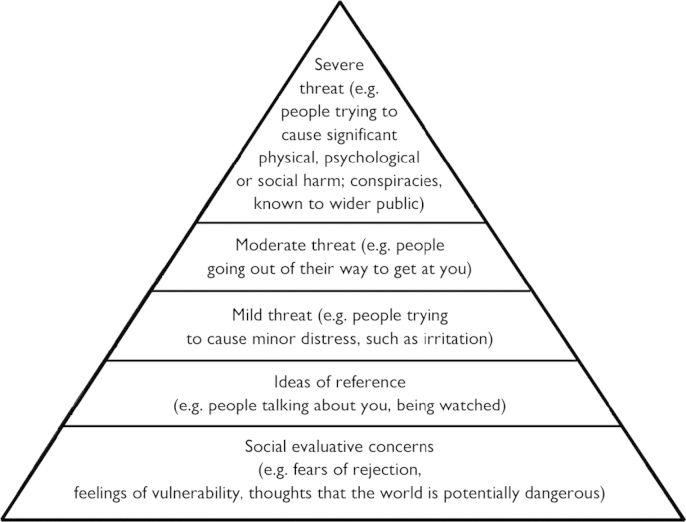
The paranoia hierarchy.

Paranoia is unfounded fear that others are trying to cause the person harm. This type of threat anticipation is hypothesised to be an extension of common feelings of vulnerability (Freeman et al., 2002). A paranoia hierarchy is conceptualised (see Fig. 1), with negative socio-evaluative concerns – the self as different and apart and hence vulnerable – underlying the experience (Freeman et al., 2005; Bebbington et al., 2013). There is empirical evidence consistent with this view. Thoughts of vulnerability to rejection in social situations predict the occurrence of paranoia (e.g., Freeman et al., 2008); ideas about the self as a failure or weak predict the persistence of persecutory delusions (e.g., Fowler et al., 2012); and negative interpersonal self concepts have been found to be associated with paranoia (e.g., Lincoln et al., 2010). This self vulnerability perspective may partly explain why levels of paranoia are higher in patients with common emotional disorders (Varghese et al., 2011).

Therefore it can be predicted that paranoia will be more likely to occur in people who view themselves negatively in comparison with other people (i.e., who perceive themselves lower in the social hierarchy). In a cross-sectional study of 1200 people, feeling inferior, less competent, and left out were associated with having paranoid thoughts (Freeman et al., 2005). Similarly, submissive behaviours, lower social rank, and self-criticism have all been associated with paranoid thinking (Allan and Gilbert, 1997; Gilbert et al., 2005; Hutton et al., 2013; Freeman et al., 2005). Social comparison may be an important factor underlying paranoia, but a causal role has not been tested.

An established correlate of social rank is height. For instance, taller people are more likely to have achieved higher educational attainment, hold jobs of higher status, have higher social esteem, earn more, and report higher levels of well-being (Jackson and Ervin, 1992; Judge and Cable, 2004; Magnusson et al., 2006; Carrieri and De Paola, 2012). Height is often regarded as conveying authority. An illustration can be seen in seating arrangements: people seeking social dominance put others in lower chairs. Therefore we tested the effect of altering height on paranoia. It was predicted that lowering an individual's height in a social situation, in comparison to his or her normal height, would lead to perceptions of lower social rank and greater levels of paranoia. It was predicted that increases in paranoia would be fully mediated by changes in social comparison.

We tested these hypotheses using immersive virtual reality (VR). An immersive VR system creates a surrounding three dimensional computer-generated world in which a person can physically move and interact with objects and virtual people. The laboratory room becomes replaced – typically via a tracked head-set worn by the person – by an alternate digitally created and computer generated world. The movements of participants are tracked in real-time so that the images are visually updated as a function of head gaze position and orientation. Testing in VR has ecological validity since it elicits responses in individuals similar to those that would occur in the real situation (Sanchez-Vives and Slater, 2005). For example, individuals who have paranoid thoughts in VR about the computer characters are more likely to report paranoid thoughts in day to day life (Freeman et al., 2008). In VR a person's height can be lowered in relation to all aspects of the environment. A study of negotiation in immersive VR found that individuals made shorter were less aggressive than individuals made taller, and the effects persisted when the negotiation task was repeated in a real face to face interaction (Yee et al., 2009).

## 2 Method

### 2.1 Participants

Sixty adult females (aged 18 or above) with paranoid thinking in the past month, but no history of severe mental illness, were tested. Participants were recruited from the general population using local radio adverts, leafleting of local areas, and posters. Interested participants completed a screening survey, which included a paranoia measure. Since there may be differences by gender in how height is perceived (e.g., Jackson and Ervin, 1992; Buunk et al., 2008; Gawley et al., 2009), we restricted recruitment in this study to one gender (women). Participants had to report persecutory thinking in the past month (a score of 17 or above) as assessed by the Paranoid Thoughts Scale Part B (Green et al., 2008). A history of severe mental illness (e.g., schizophrenia) or substance dependence was an exclusion criterion. A history of being diagnosed or treated for other mental health problems was noted; 15 participants reported a history of mental illness, predominately depression.

### 2.2 Assessments

Demographic information was collected from participants and their height measured.

#### 2.2.1 Paranoid thoughts scale part B (GPTS-B) (Green et al., 2008)

The GPTS-Part B measures persecutory ideation, as defined by Freeman and Garety (2000), over the past month. Each of the sixteen items in the scale (e.g., ‘Certain individuals have had it in for me’ ‘People have been hostile towards me on purpose’ ‘I was sure someone wanted to harm me’ ‘I was convinced there was a conspiracy against me’) are rated by the person on a 5-point scale (1–5). Scores can range from 16 to 80, with 16 indicating the absence of persecutory ideation and higher scores indicating greater persecutory ideation.

#### 2.2.2 State social paranoia scale (SSPS) (Freeman et al., 2007)

The SSPS was specifically designed to assess paranoia in VR. It comprises ten persecutory items (e.g., ‘Someone stared at me in order to upset me’; ‘Someone was trying to isolate me’; ‘Someone had it in for me’; ‘Someone was trying to make me distressed’), each rated on a 5-point scale. Higher scores on the scale indicate greater levels of persecutory thinking.

#### 2.2.3 Social comparison scale (Allan and Gilbert, 1995)

This measure comprises eleven bipolar scales: inferior–superior, incompetent–competent, unlikeable–likeable, left out–accepted, different–same, untalented–more talented, weaker–stronger, unconfident–more confident, undesirable–more desirable, unattractive–more attractive, outsider–insider. Each is rated on a 1–10 scale. Higher scores indicate a more positive view of the self in relation to others. In the current study the participants were asked to rate the scale for how they felt in relation to the computer characters on the tube. The internal reliability of the scale in the current scale was high (Cronbach alpha (normal height)=0.89, Cronbach alpha (lowered height)=0.93).

### 2.3 Virtual reality

**Fig. 2:**
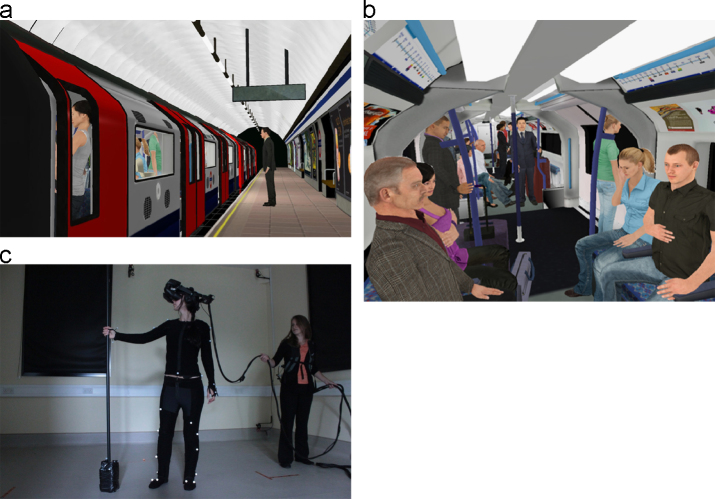
(a) The virtual train at the station. (b) The virtual train carriage. (c) A participant in the virtual reality laboratory.

The experiment was conducted using an immersive virtual environment, which displayed a London underground tube station and a train journeying between stations. The equipment and environment was a substantial up-grade on that used by Freeman et al. (2008). Images of the lab and scenario can be seen in Fig. 2.

#### 2.3.1 Equipment

The virtual underground was displayed using an NVIS SX111 head mounted display (HMD). The HMD has a wide field of view (111°), provides an image offset for the centre of projection for each eye at the rate of 60 hz, and provides headphones to render audio. The user's head position was tracked using an Intersense IS900 tracking device. The head tracking data was read by a VRPN server application and sent to a VRPN client dll used by the XVR application.

#### 2.3.2 Environment

The model of the station and the tube car were built using Maya. It consisted of a virtual platform and virtual tube train cars. The lighting was baked into the textures. The model was exported from 3dsmax to the aam format. The model was rendered using the XVR application platform. The XVR application software was run on a Windows XP machine with an Intel Core i7 CPU with 4 GB of RAM. The machine had an Nvidia GTX580 graphics card. The avatars used to implement the virtual people in the scenario were Rocket Box avatars. The avatars were exported from 3dsmax to the cal3d format. The animations used to display the virtual characters were captured using an Arena OptiTrack motion capture system. The animations were edited in MotionBuilder and exported to caf files via 3dsmax. The motions were applied to the avatars using the Hardware Accelerated Library for Character Animation (HALCA). The avatars in the study were responsive to whether the participant was in their field of view and if the gaze of the participant was directed at a particular avatar. Each avatar was programmed with a factor, varying between 1.5 and 3 s, which determined how long the participant could gaze at the avatar before the avatar would look back. The length of the look of the avatar was a function of the participant's distance; the maximum look was 2 s when the participant was over 2 m away from the avatar and the minimum look length was 0.5 s when the participant was less than 0.5 m away.

Participants began on the station platform, before the train arrived. Once the train was present they entered the train carriage. The doors closed and a journey of 6 min occurred, including stops at stations. The carriage in which the participant stood was populated by 23 avatars (11 males and 12 females). A soundtrack of a tube journey, including low level conversation was played. For each condition an identical journey occurred. However in the lowered height condition the participant was reduced in height in the virtual tube by 25 cm, equivalent to approximately the height of a head.

### 2.4 Procedure

The study received approval from the University of Oxford's research ethics committee. Upon arrival for testing participants completed the baseline measures, had a short break, and were then randomised to receive either the normal or lowered height VR condition first. Upon completion of each VR train ride, the measures of paranoia and social comparison in VR were completed. There was a 5 min break between the two VR tests. Participants were not told until the end of the experiment that their height had been altered in VR. They were simply told: ‘you will be going in to virtual reality twice. After each time we will ask you to complete questionnaires to tell us about your reactions and thoughts about it.” At the end of the experiment the participants were asked whether they had noticed any difference between the two VR experiences.

### 2.5 Analysis

Data from this two-condition two-period crossover trial were analysed using Stata version 12 (StataCorp, 2011). There were no missing data. Although these data could be analysed through a repeated measures analysis of variance, a simpler but mathematical equivalent procedure is to look at a simple linear regression model to evaluate the effect of order of the presentation of the two VR conditions on the *change* in the outcome variable from period one to period two (or, more accurately, the change score divided by half so that the resulting regression coefficient is interpreted as the effect of the experimental condition (lowering) on outcome). The logic and procedure for doing this is explained by Armitage et al. (2002). Significance test results for all the analyses are quoted as two-tailed probabilities.

If an effect of shortening on both self comparison with other people (the putative mediator) and level of paranoia (the final outcome) is demonstrated then the above analysis has the advantage that it can be adapted to evaluate mediation using the strategy suggested by Baron and Kenny (1986), This involves three stages: (1) regression of change in self comparison on VR order, (2) regression of change in paranoia on VR order and (3) regression of change in paranoia on VR order together with change in self comparison.

## 3 Results

### 3.1 Demographic information

The participant group had an average age of 31.5 (S.D.=13.0, minimum=18, maximum=62). The average height was 166.5 cm (S.D.=6.7, minimum=153.0, maximum=193.0). The ethnicity of the group was White (*n*=47), Black African (*n*=4), Indian (*n*=2), Pakistani (*n*=2), Chinese (*n*=1), other (*n*=4). The highest level of education reached was none/GCSE (*n*=4), A-level (*n*=23), degree (*n*=16), postgraduate degree (*n*=17). The mean score of the participant group on the Paranoid Thoughts Scale Part B was 25.6 (S.D.=11.5, minimum=17, maximum=63, 25 percentile=18.0, 50th percentile=19.5, 75th percentile=28.8).

### 3.2 Social comparison

Scores for social comparison in the normal and lowered height conditions are shown in Table 1. It can be seen that mean scores were almost eight points lower in the reduced height condition compared to the normal height condition. This was confirmed statistically. Order of VR height presentation was a significant predictor of social comparison change scores, coefficient=−7.8 (95% confidence interval=−10.1, −5.6), standard error=1.12, *t*=−6.99, *p*<0.001.

### 3.3 Paranoia in VR

Scores for paranoia in the normal and lowered height conditions are shown in Table 2. It can be seen that mean scores on the SSPS were two points higher in the lowered height condition compared to the normal height condition. This was confirmed statistically. Order of VR height presentation was a significant predictor of paranoia change scores, coefficient=2.0 (95% confidence interval=0.4, 3.6), standard error=0.82, *t*=2.44, *p*=0.018. A selection of participant comments about the experiences is presented in Table 3.

### 3.4 Test of mediation

The three stages for the test of mediation by social comparison of the relationship between height and paranoia are presented in Table 4. Consistent with the previous analyses, it can be seen that VR order of height presentation predicted change in social comparison scores (stage 1) and also change in paranoia scores (stage 2). However VR order of height presentation was not a significant predictor of change in paranoia scores when social comparison change scores were also entered (stage 3). The results show that social comparison fully mediates the relationship between height and paranoia.

## 4 Discussion

Height is associated with perceptions of dominance (Sorokowski, 2010). Our hypothesis was that lowering a person's height would lead to a reduction of status in a social situation. A consequence would be the occurrence of paranoia, building upon the sense of greater vulnerability. Immersive virtual reality provided the means to control a person's height in relation to all aspects of an environment. We lowered the participants' height by around a head. Mostly this was not consciously noticed by participants. The results were very clear: lowering of height led to more negative evaluations of the self compared with others and greater levels of paranoia. The changes in paranoia were fully mediated by the changes in social comparison (assuming that there are no common causes arising after randomisation – i.e., no hidden confounding – the crossover design copes with all pre-randomisation confounders). The study provides a strong demonstration of the importance of social status to the occurrence of paranoid fears. It supports the theoretical idea that paranoia builds upon perceived vulnerabilities of the self.

This is the first study of height, paranoia, and social comparison. It can be developed in a number of ways. We restricted the sample to one sex. Clearly it would be of interest to carry out the same study in males. Arguably height changes would produce greater effects in men, since, for instance, men and women over-report their own height but men do this to a greater extent than women (Spencer et al., 2002). There is also the interesting test of whether increasing height lowers paranoia, and whether these manipulations affect patients with persecutory delusions. Other ways to manipulate social status would also be possible in VR, for instance the use of optical markers on participants enables them to be given virtual bodies and these can be altered to either increase or decrease confidence. Overall, this line of enquiry can provide important causal evidence of the contribution of socio-cognitive processes to the occurrence of paranoid fears. Clinically, these types of manipulations could be used to increase an individual's self-confidence in social situations and hence lower paranoia.

## Figures and Tables

**Table 1:** Social comparison in VR.

Height	Order	Mean VR social comparison	S.D.
Normal	Normal then lowered	58.9	15.1
	Lowered then normal	61.0	10.4
	Total	60.0	12.9

Lowered	Normal then lowered	50.6	16.8
	Lowered then normal	53.7	10.7
	Total	52.1	14.1

**Table 2:** Paranoia in VR.

Height	Order	Mean VR paranoia	S.D.

Normal	Normal then lowered	13.0	6.5
	Lowered then normal	11.1	2.2
	Total	12.1	4.9

Lowered	Normal then lowered	15.5	9.6
	Lowered then normal	12.6	4.8
	Total	14.1	7.7

**Table 3:** Selection of participant comments.

“It felt different in the two times. I felt more vulnerable the first time [lowered condition], and also the man with the legs in the aisle was acting in a hostile way towards me the first time, but I did not feel it so much the second time, even though his legs were in the same place, I do not know why!” [Did not notice the height difference].
“I felt like people were staring more the first time [lowered condition], the second time just felt more comfortable but I do not know why.” [Did not notice the height difference].
“I felt more intimidated the first time [lowered condition], not sure why. There was a girl who kept putting her hand to her face, the man with the blue t-shirt was shaking his head at me, they were staring more at me.” [Did not notice the height difference].
“There were differences between the two times, like the people were moving differently, and there were suitcases one time but not the other.” [Did not notice the height difference].
“I noticed the second time I was shorter. People, even suitcases, were feeling high. I was frustrated to feel like a child again, felt out of place on the tube, because I was not an adult.”
“Yes I noticed feeling shorter the second time. It felt more comfortable then because I was not in the line of eye sight and did not catch people’s eyes.”
“Maybe I did notice a difference in my height, but I thought it was just that the headset had been put on differently so I took no notice of it.”

**Table 4:** Analysis of mediation.

	Coefficient	Standard error	*t*	*p*-value	95% C.I.
1. *Dependent variable: Social comparison change*					
VR height order	−7.83	1.12	−6.99	<0.001	−10.08, -5.99
2. *Dependent variable: Paranoia change*					
VR height order	2.00	0.82	2.44	0.018	0.36, 3.65
3. *Dependent variable: Paranoia change*					
Social comparison change	−0.24	0.09	−2.61	0.011	−0.42, −0.06
VR height order	0.12	1.06	0.12	0.907	−2.00, 2.25

## References

[bib1] Allan S., Gilbert P. (1997). Submissive behaviour and psychopathology. British Journal of Clinical Psychology.

[bib2] Allan S., Gilbert P. (1995). Social comparison scale: psychometric properties and relationship to psychopathology. Personality and Individual Differences.

[bib3] Armitage P., Berry G., Matthews J.N.S. (2002). Statistical Methods in Medical Research.

[bib4] Baron R., Kenny D. (1986). The moderator-mediator variable distinction in social psychological research. Journal of Personality and Social Psychology.

[bib5] Bebbington P., McBride O., Steel C., Kuipers E., Brugha T., Jenkins R., Meltzer H., Freeman D. (2013). The structure of paranoia in the general population. British Journal of Psychiatry.

[bib6] Buunk A., Park J., Zurriaga R., Klavina L., Massar K. (2008). Height predicts jealousy differently for men and women. Evolution and Human Behavior.

[bib7] Carrieri V., De Paola M. (2012). Height and subjective well-being in Italy. Economics and Human Biology.

[bib8] Fowler D., Hodgekins J., Garety P., Freeman D., Kuipers E., Dunn G., Smith B., Bebbington P. (2012). Negative cognition, depressed mood, and paranoia. Schizophrenia Bulletin.

[bib9] Freeman D., Garety P.A. (2000). Comments on the content of persecutory delusions: Does the definition need clarification?. British Journal of Clinical Psychology.

[bib10] Freeman D., Garety P.A., Bebbington P.E., Smith B., Rollinson R., Fowler D., Kuipers E., Ray K., Dunn G. (2005). Psychological investigation of the structure of paranoia in a non-clinical population. British Journal of Psychiatry.

[bib11] Freeman D., Garety P., Kuipers E., Fowler D., Bebbington P. (2002). A cognitive model of persecutory delusions. British Journal of Clinical Psychology.

[bib12] Freeman D., Pugh K., Antley A., Slater M., Bebbington P., Gittins M., Dunn G., Kuipers E., Fowler D., Garety P. (2008). A virtual reality study of paranoid thinking in the general population. British Journal of Psychiatry.

[bib13] Freeman D., Pugh K., Green C., Valmaggia L., Dunn G., Garety P. (2007). A measure of state persecutory ideation for experimental studies. Journal of Nervous and Mental Disease.

[bib14] Gawley T., Perks T., Curtis J. (2009). Height, gender, and authority status at work. Sex Roles.

[bib15] Gilbert P., Boxall M., Cheung M., Irons C. (2005). The relation of paranoid ideation and social anxiety in a mixed clinical population. Clinical Psychology and Psychopathology.

[bib16] Green C., Freeman D., Kuipers E., Bebbington P., Fowler D., Dunn G., Garety P. (2008). Measuring ideas of persecution and social reference. Psychological Medicine.

[bib17] Hutton P., Kelly J., Lowens I., Taylor P., Tai S. (2013). Self-attacking and self-reassurance in persecutory delusions. Psychiatry Research.

[bib18] Jackson L., Ervin K. (1992). Height stereotypes of women and men. Journal of Social Psychology.

[bib19] Judge T., Cable D. (2004). The effect of physical height on workplace success and income. Journal of Applied Psychology.

[bib20] Lincoln T.M., Mehl S., Ziegler M., Kesting M.-L., Exner C., Reif W. (2010). Is fear of others linked to an uncertain sense of self? The relevance of self-worth, interpersonal self-concepts, and dysfunctional beliefs to paranoia. Behavior Therapy.

[bib21] Magnusson P., Rasmussen F., Gyllensten U. (2006). Height at age 18 years is a strong predictor of attained education later in life. International Journal of Epidemiology.

[bib22] Sanchez-Vives M., Slater M. (2005). From presence to consciousness through virtual reality. Nature Reviews. Neuroscience.

[bib23] Sorokowski P. (2010). Politicians’ estimated height as an indicator of their popularity. European Journal of Social Psychology.

[bib24] Spencer E., Appleby P., Davey G., Key T. (2002). Validity of self-reported height and weight in 4808 EPIC-Oxford participants. Public Health Nutrition.

[bib25] StataCorp (2011). Stata Statistical Software. Release 12.

[bib26] Varghese D., Scott J., Welham J., Bor W., Najman J., O’Callaghan M., Williams G., McGrath J. (2011). Psychotic-like experiences in major depression and anxiety disorders. Schizophrenia Bulletin.

[bib27] Yee N., Bailenson J., Ducheneaut N. (2009). The Proteus effect: implications of transformed digital self-representation on online and offline behavior. Communication Research.

